# Supplementary material for: Risk Factors for Long-Term Nutritional Disorders One Year After COVID-19: A Post Hoc Analysis of COVID-19 Recovery Study II
Source: Nutrients. 2024 Dec 7;16(23):4234. doi: 10.3390/nu16234234 (PMC11644535; doi:10.3390/nu16234234)
Supplement: Supplementary file 1 [file nutrients-16-04234-s001.zip › nutrients-3333990-supplementary.pdf]

Table S1. Subjective recovery and Breathlessness and Fatigue items.

Score	Subjective Recovery	Breathlessness	Fatigue
0	No recovery at all.	I only get breathless with strenuous exercise	Fully active, able to carry on all pre-disease performance without restriction
1	No awareness of recovery.	I get short of breath when hurrying on level ground or walking up a slight hill	Restricted in physically strenuous activity but ambulatory and able to carry out work of a light or sedentary nature, e.g., light house work, office work
2	Neither recovering nor not recovering.	On level ground, I walk slower than people of my age because of breathlessness, or I have to stop for breath when walking at my own pace on the level	Ambulatory and capable of all selfcare but unable to carry out any work activities; up and about more than 50% of waking hours
3	Feeling recovered.	I stop for breath after walking about 100 yards or after a few minutes on level ground	Capable of only limited selfcare; confined to bed or chair more than 50% of waking hours
4	Feeling very much recovered.	I am too breathless to leave the house or I am breathless when dressing/undressing	Completely disabled; cannot carry on any selfcare; totally confined to bed or chair
